# Supplementary figures and images for: BROCKMAN: deciphering variance in epigenomic regulators by k-mer factorization
Source: BMC Bioinformatics. 2018 Jul 3;19:253. doi: 10.1186/s12859-018-2255-6 (PMC6029352; doi:10.1186/s12859-018-2255-6)

Figure S1

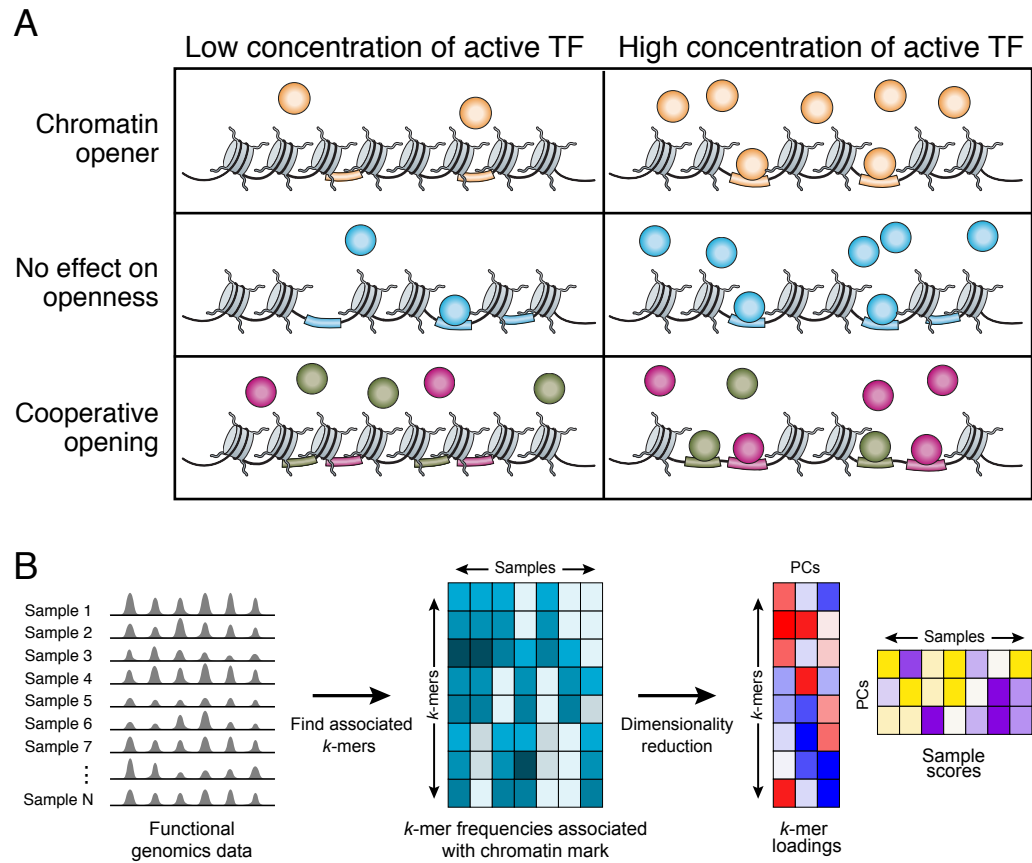

Supplement: Supplementary file 1 — Figure S1. BROCKMAN computational pipeline. A bash pipeline and other computational resources are available on GitHub (https://carldeboer.github.io/brockman.html). Tools/functions used for each step are indicated in brackets. (PDF 545 kb) [file 12859_2018_2255_MOESM1_ESM.pdf]

Figure S2

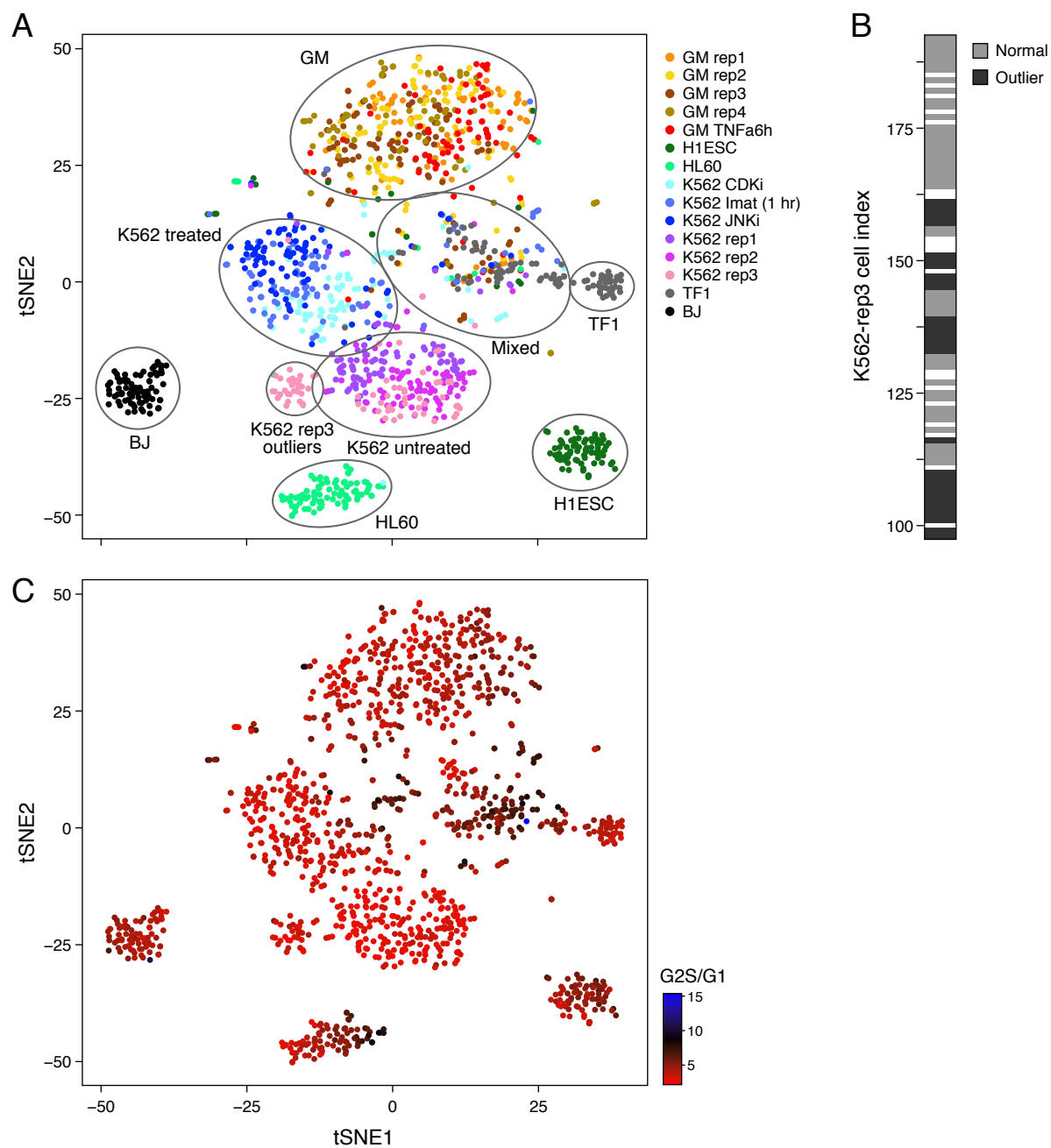

Supplement: Supplementary file 2 — Figure S2. PCs that distinguish replicates. Shown are the Bonferroni-corrected P-values (y axis) and AUROC values (x axis) for how well each PC separates each untreated K562 replicate from the other two replicates. Colors indicate the replicate being compared to the other two. Red horizontal line: P-value cutoff (0.1) below which PCs were considered to separate batches.) (PDF 185 kb) [file 12859_2018_2255_MOESM2_ESM.pdf]

# Figure S3

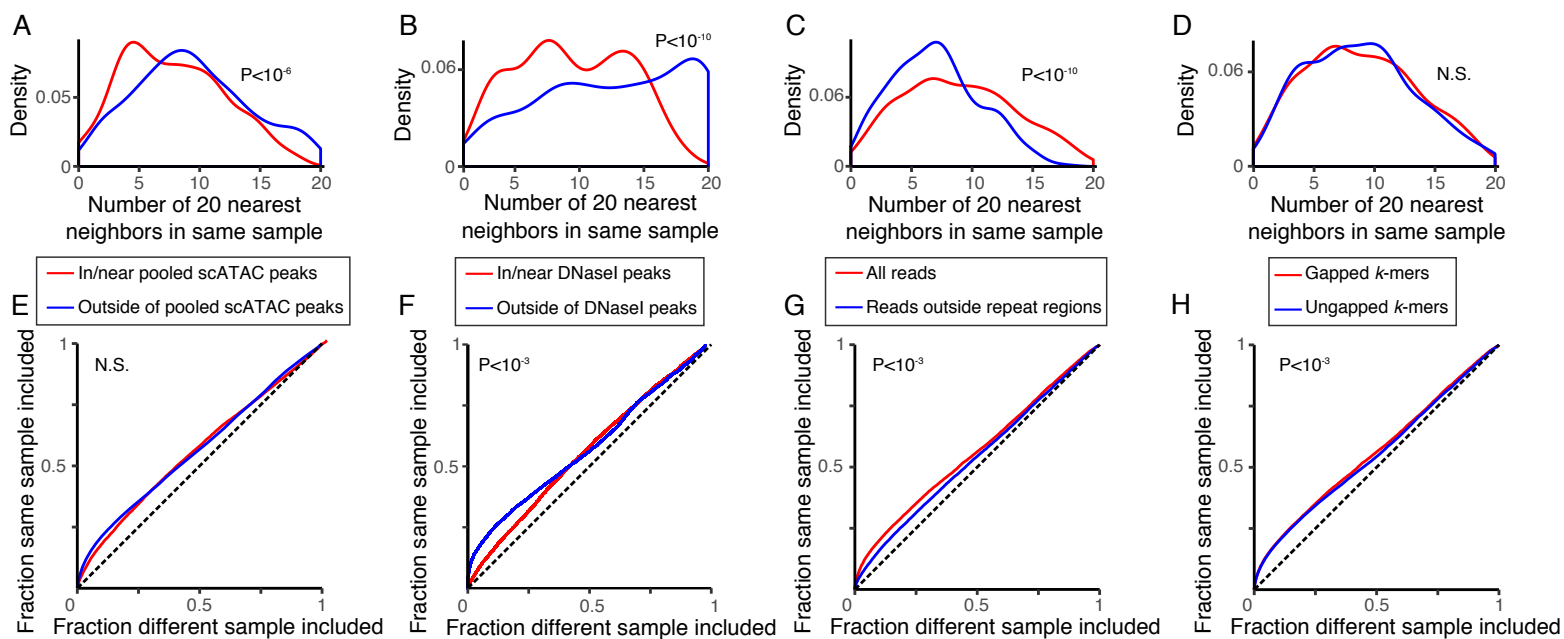

Supplement: Supplementary file 3 — Figure S3. The TFs enriched in PCs have lower expression. A, B) CDF of the mean (population) expression (A, x axis) or mean-corrected CV (B, x axis; Methods) for the most (blue) and least (pink) significant TFs enriched in the PCs from a BROCKMAN analysis of untreated K562 cells. C) The relationship between the mean expression (x axis) and CV (y axis) for all genes in WT K562 data (dots). Names of TFs with the highest mean-corrected CV are labeled and AP-1 factors are bolded. Pink, blue: TFs with least and most significant PC enrichment. (PDF 200 kb) [file 12859_2018_2255_MOESM3_ESM.pdf]

Figure S4

A

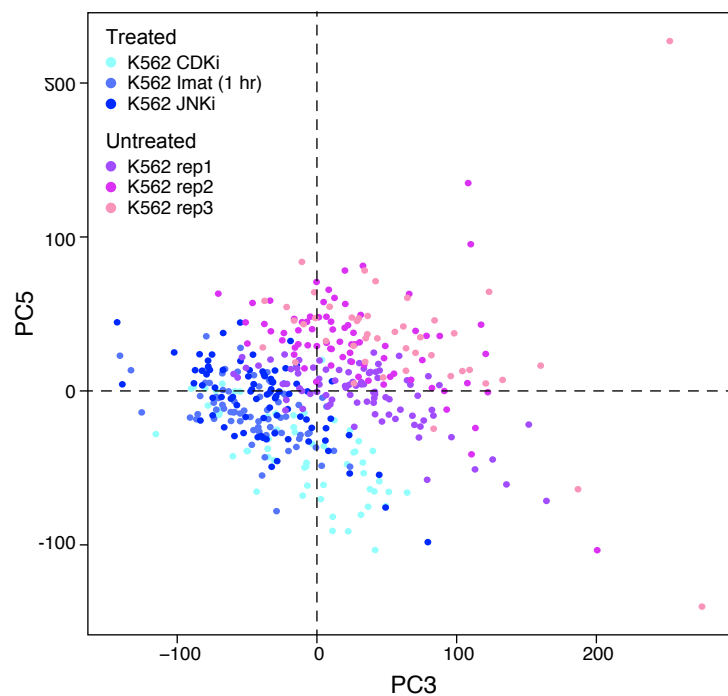

B

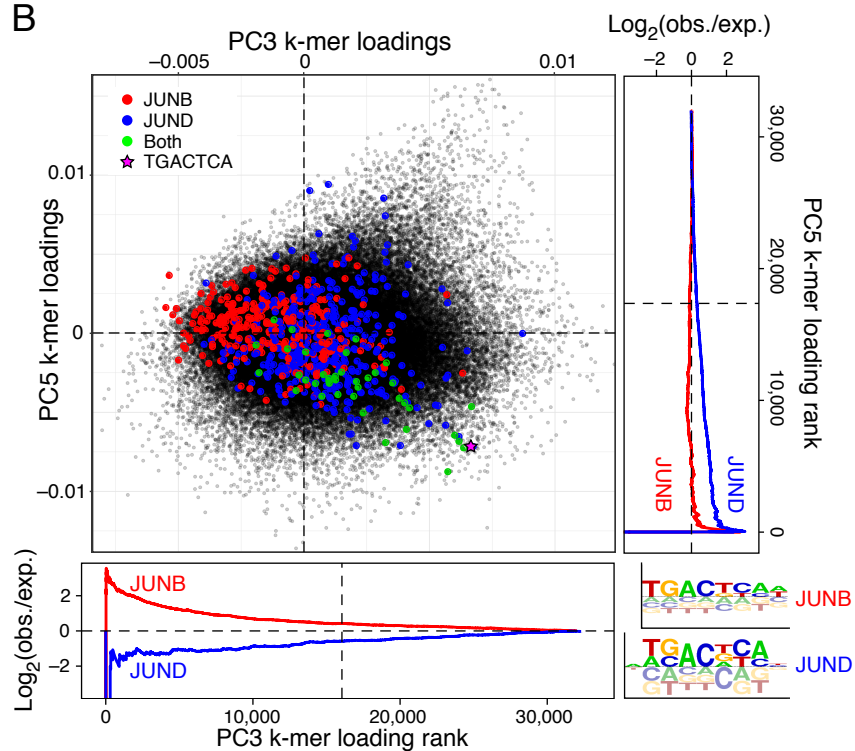

Supplement: Supplementary file 5 — Figure S4. Cooperativity between TFs results in steeper binding curves. The predicted fractional TF occupancy (y axis) for a given concentration of the TF (x axis), when the concentration of the cooperatively-interacting TF is constant. The two binding curves are aligned at 50% occupancy to emphasize the differences in the slopes. Modeling was done as described in Methods. (PDF 1969 kb) [file 12859_2018_2255_MOESM5_ESM.pdf]
