# Supplementary material for: BROCKMAN: deciphering variance in epigenomic regulators by k-mer factorization
Source: BMC Bioinformatics. 2018 Jul 3;19:253. doi: 10.1186/s12859-018-2255-6 (PMC6029352; doi:10.1186/s12859-018-2255-6)
Supplement: Supplementary file 4 — Table S1. Summary of TFs associated with the different untreated K562 cell-variable PCs. TFs are listed in decreasing order of enrichment significance, with TFs filtered for redundancy between motifs as described in the Methods. Interacting TFs are not indicated and examples given in the text are for illustrative purposes. (DOCX 16 kb) [file 12859_2018_2255_MOESM4_ESM.docx]

**Table S1: Summary of TFs associated with the different untreated K562 cell-variable PCs.** TFs are listed in decreasing order of enrichment significance, with TFs filtered for redundancy between motifs as described in the **Methods**. Interacting TFs are not indicated and examples given in the text are for illustrative purposes.

| PC | TFs enriched in highly weighted *k*-mers | TFs enriched in lowly weighted *k*-mers |
| --- | --- | --- |
| PC3 | RUNX2, RREB1, TE­RF2, SMARCC1, ZNF524, KLF3, SREBF1, TBX15, TWIST1 | TBP, FOXD2, E2F2, POU4F1, NFATC1, IRF9, HOXD13, MEF2C, STAT5B, ZNF384, TEAD3, CDC5L, FOXP1, YY1, E2F4, LCOR, SOX12, FUBP1, SPI1, PRDM4, BBX, MLL, HES2, E2F1, HHEX, SP6, CIC |
| PC5 |  |  |
| PC6 | BATF, NFE2L2, TGIF1, ATF3 |  |
| PC7 | IRF2, SPI1, SMARCC1, ELF1, SPIB, IRF9, STAT1 | HIC2, ZNF740, KLF1, ZNF143, MZF1, HOXB4 |
| PC13 | NFYA, CTCF | JUNB, SNAI3, MAFF, SMAD3 |
| PC14 | MZF1, ZNF740, GATA1, CREB1 |  |
| PC26 | JUN, RUNX2 |  |
| PC27 | ESRRA |  |
